# Supplementary material for: Inflammatory monocytes promote pre-engraftment syndrome and tocilizumab can therapeutically limit pathology in patients
Source: Nat Commun. 2021 Jul 6;12:4137. doi: 10.1038/s41467-021-24412-1 (PMC8260612; doi:10.1038/s41467-021-24412-1)
Supplement: Supplementary file 1 — Supplementary information [file 41467_2021_24412_MOESM1_ESM.pdf]

Supplementary Information

Inflammatory Monocytes Promote Pre-Engraftment Syndrome Depending on IL-6

Jin et al.

Supplementary Information contains

Supplementary Figures 1–8

Supplementary Table 1-7

Consort diagram

Supplementary Note 1-2

Supplementary Figures

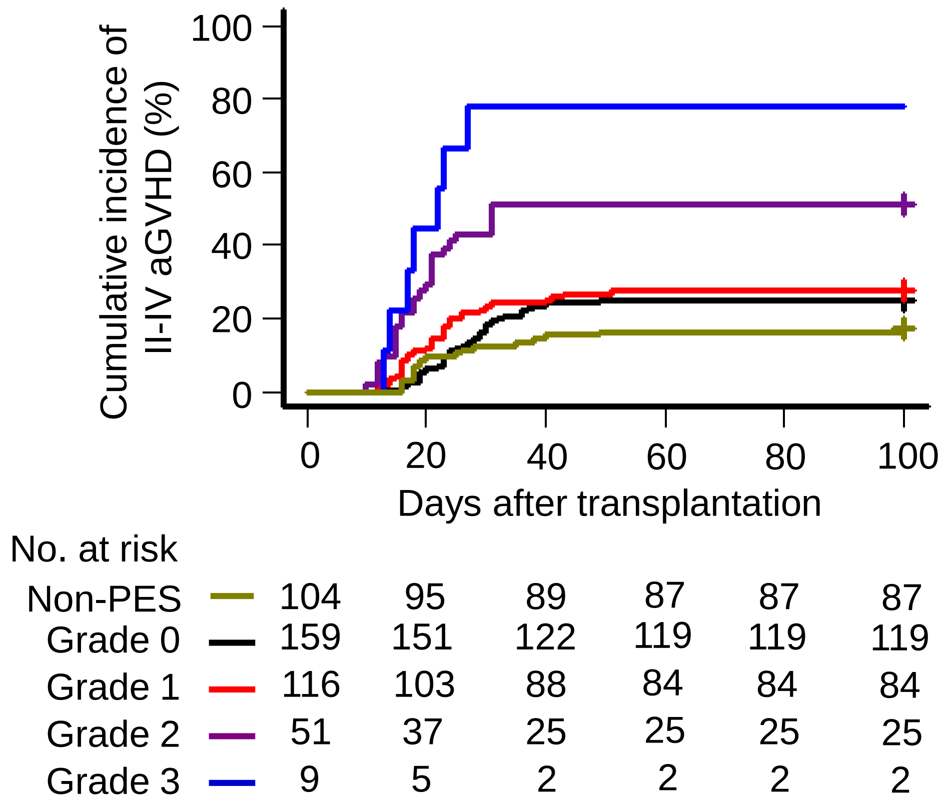

Supplementary Fig. 1 The cumulative incidence of grades II–IV acute graft-versus-host disease on day 100 in patients with different PES grades. The number of patients at risk for II–IV acute graft-versus-host disease is listed below each time point. Source data is provided as a Source Data file.

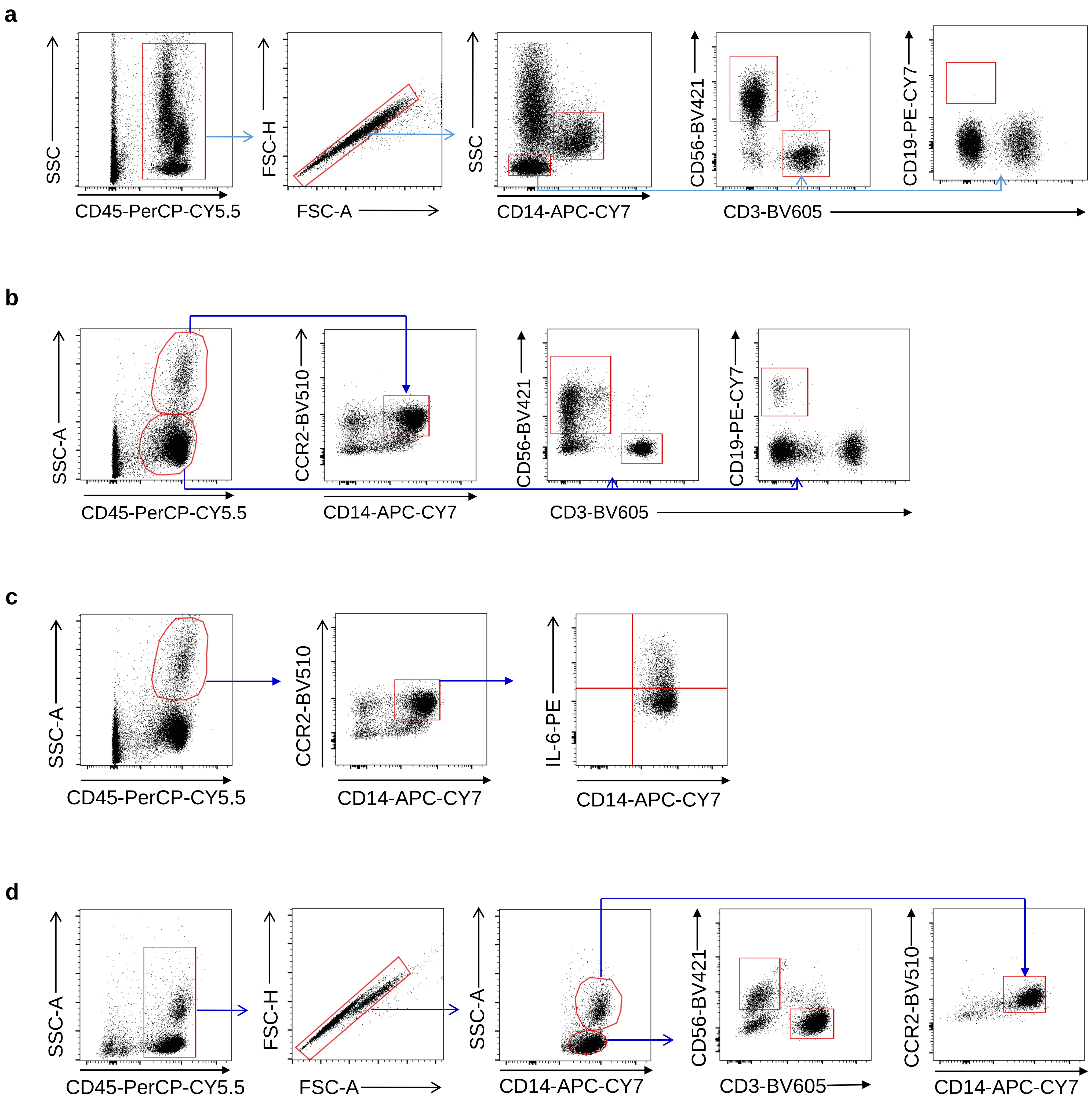

Supplementary Fig. 2 Gating strategies used for FACS analysis. (a) Gating strategy presented on Fig. 2b,c and d. (b) Gating strategy presented on Fig. 3h, i. (c) Gating strategy presented on Fig. 4a. (d) Gating strategy presented on Fig. 4g.

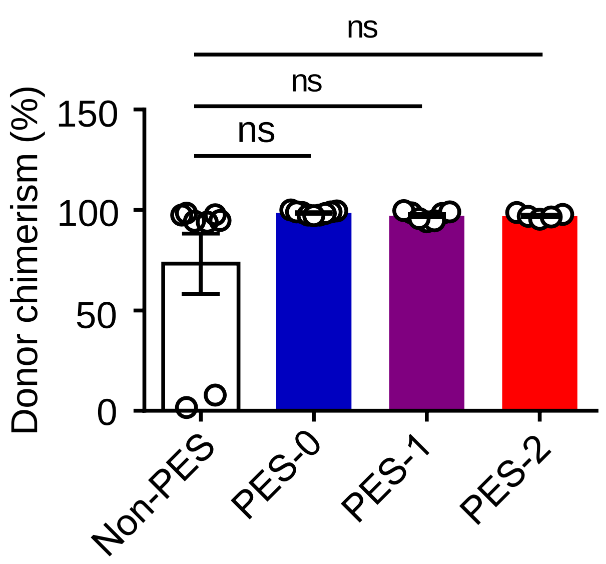

Supplementary Fig. 3 Donor chimerism in the peripheral blood of recipients on day 14 post UCBT (n=8, 9, 7, 5, respectively). Each symbol represents an independent individual. Data are presented as mean ± SEM. One-way ANOVA, n.s., not significant. Source data is provided as a Source Data file.

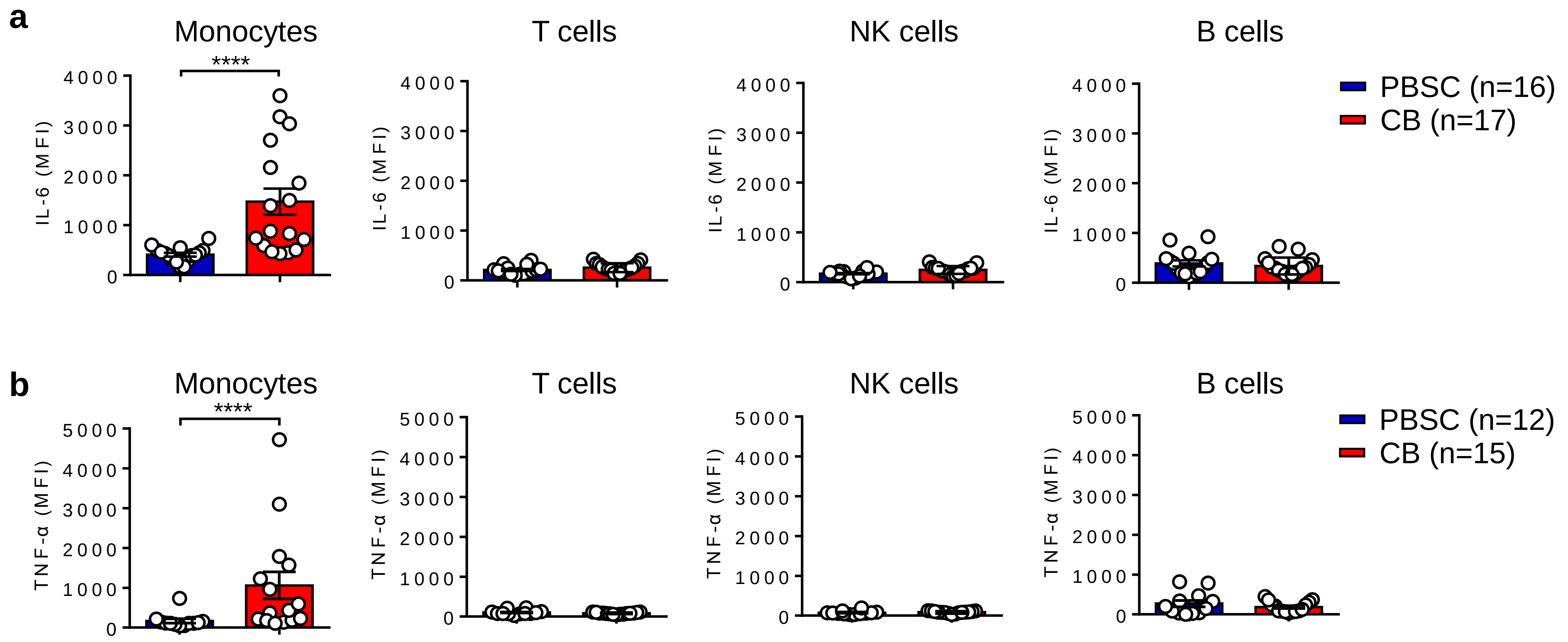

Supplementary Fig. 4 Inflammatory cytokines are high-expression in monocytes derived from cord blood. (a) Statistical data generated by the MFI of IL-6 from mononuclear cells from peripheral blood stem cells (PBSC) or cord blood (CB). n = 16 and 17, respectively. The MFI of IL-6 from CB monocytes is higher than that from PBSC monocytes (p=1.9e-5). (b) Statistics calculated by the MFI of TNF-α from mononuclear cells from PBSC or CB. n = 12 and 15, respectively. The MFI of TNF-α from CB monocytes is higher than that from PBSC monocytes (p=2.3e-4). Data are presented as mean ± SEM. Mann-Whitney test (two-sided). ****$p < 0.0001$. Source data is provided as a Source Data file.

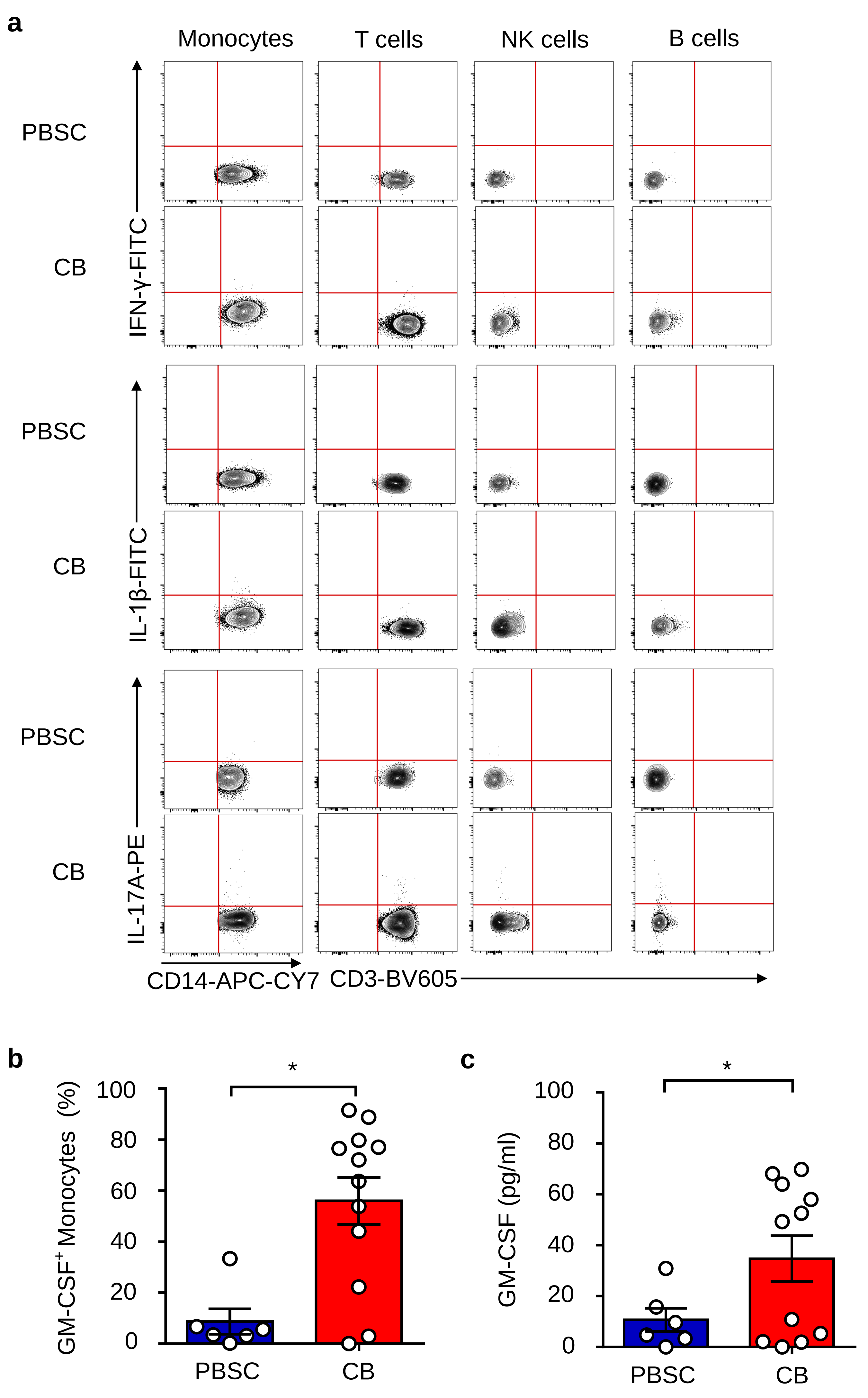

Supplementary Fig. 5 Expression of inflammatory cytokines in monocytes. (a) Frequency of IFN-γ-producing cells, IL-1β-producing cells and IL-17A-producing cells within the $CD45^{+}$ population from peripheral blood stem cells as well as cord blood mononuclear cells and cultured for 6 h. Representative plots are shown from a total of seven independent experiments. (b) Percentage of monocytes in GM-CSF^{+} cells obtained from peripheral blood stem cells and cord blood mononuclear cells. Each data point represents a biologically-independent sample. n = 6 and 12, respectively. Mann-Whitney test (two-sided) (p=0.0241). (c) ELISA of GM-CSF in supernatants from peripheral blood stem cells or cord blood mononuclear cells and cultured for 6 h. n = 6 and 11, respectively. Mean difference=23.99; 95% CI: 2.158-45.82; Unpaired t-test with Welch's correction (two-sided) (p=0.0335). PBSC, peripheral blood stem cells. CB, cord blood mononuclear cells. Each data point represents a biologically-independent sample. Data in (b) and (c) are presented as mean ± SEM. $*p < 0.05$. Source data is provided as a Source Data file.

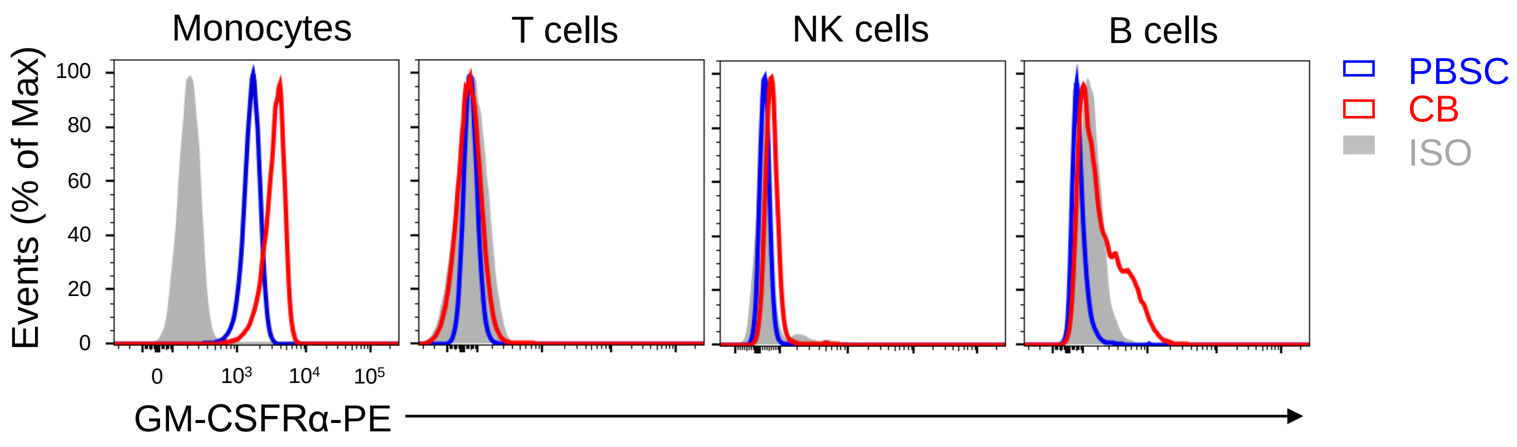

Supplementary Fig. 6 Cord blood monocytes express higher levels of GM-CSFRα. Representative histograms of GM-CSFRα in monocytes, T cells, NK cells, and B cells from peripheral blood stem cells or cord blood; n=9 for peripheral blood stem cells; n=11 for cord blood. Source data is provided as a Source Data file.

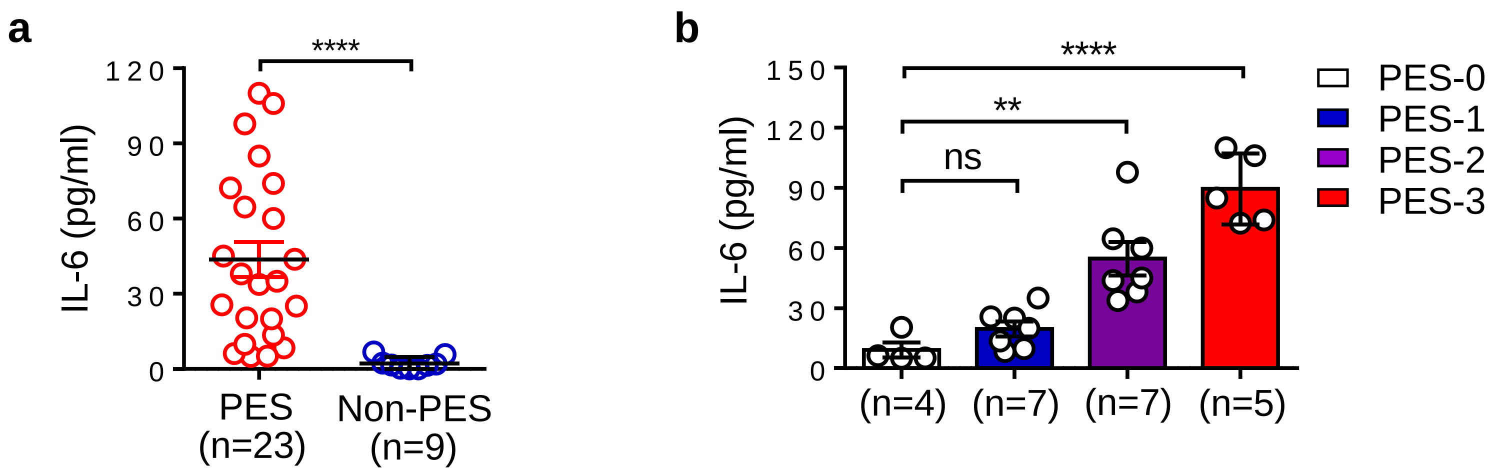

Supplementary Fig. 7 The severity of PES is highly correlated with IL-6 levels. (a) Plasma IL-6 levels were significantly higher in patients with PES than those without PES. Mean difference=-41.42, 95% CI: -56.08 to -26.76; t-test (two-sided); p = 6e-6. (b) Plasma IL-6 levels were significantly higher in patients with severe PES. One-way ANOVA (PES-1 vs. PES-0: mean difference=10.44, 95% CI: -10.61 to 31.49, p=0.312; PES-2 vs. PES-0: mean difference=45.51, 95% CI: 24.46 to 66.56, p=2.31e-4; PES-3 vs. PES-0: mean difference=80.26, 95% CI: 57.73 to 102.78, p=4.681e-7). Data are presented as mean ± SEM. n.s., not significant, $**p < 0.01$, $****p < 0.0001$. Source data is provided as a Source Data file.

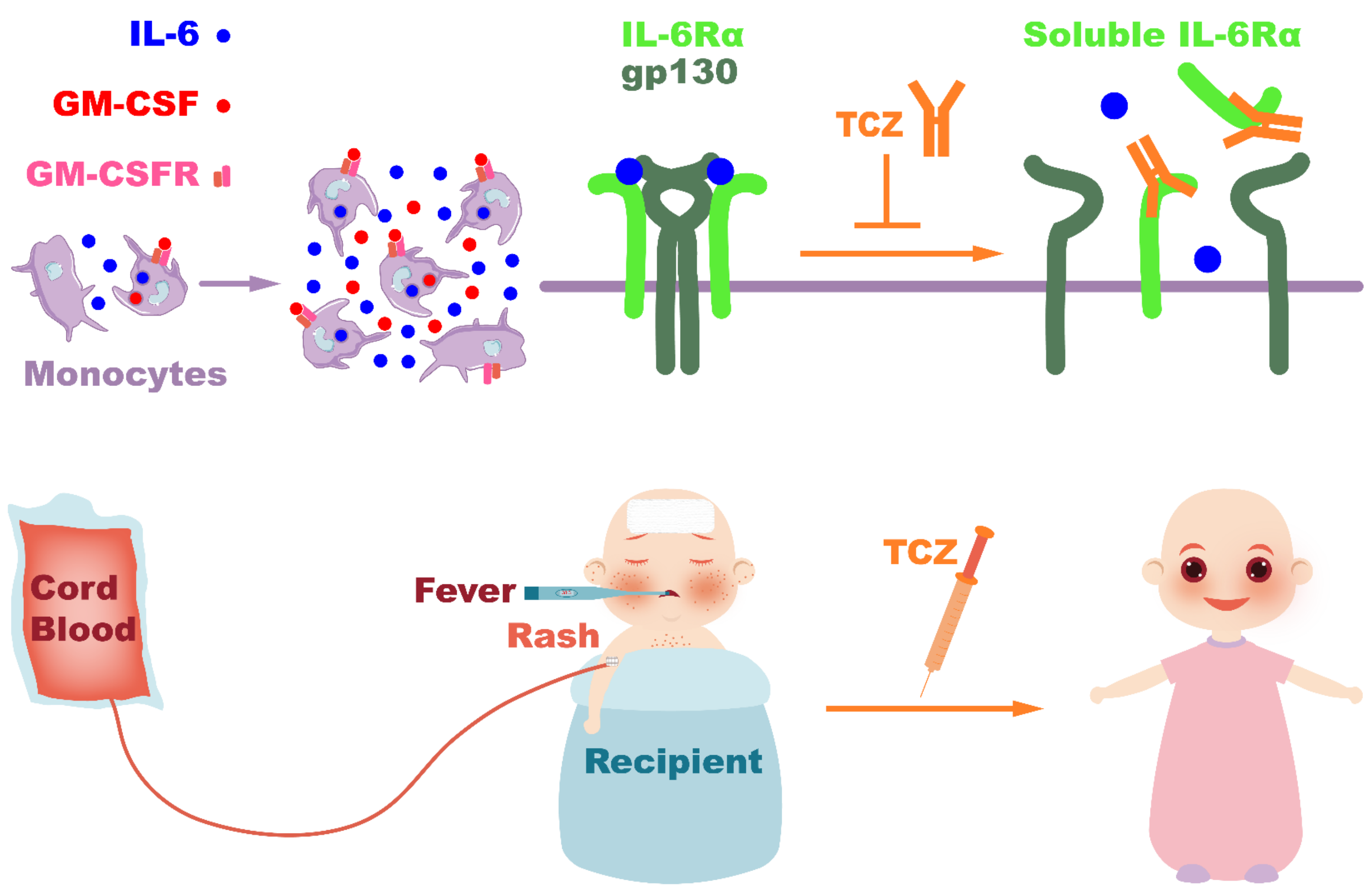

Supplementary Fig. 8 Diagram of cytokine dysregulation in PES. Monocytes derived from cord blood possess inflammatory characteristics, and secrete both GM-CSF and IL-6. Cord blood monocytes expressed high levels of GM-CSFR and responded to GM-CSF with high levels of IL-6 secretion. Subsequently, these cells undergo rapid expansion in the recipient. Levels of both GM-CSF and IL-6 were increased in the sera of PES patients. Intervention with tocilizumab (TCZ), the monoclonal antibody that targets the IL-6 receptor, is an effective treatment for patients with refractory PES. We created this schematic diagram using Adobe Illustrator CS6.

Supplementary Table 1: Grading system used for pre-engraftment syndrome (PES).

Risk factors	
1. Symptoms occur < 7 days after UCBT*<br>2. More than two symptoms (rash, diarrhea, abdominal pain, hypoxia, cough, edema)<br>3. Non-responsive to corticosteroids	
Grading system	
Grade 0	Lack of a risk factor; symptoms are minor and mild; responds quickly to corticosteroids
Grade 1	Presence of one risk factor
Grade 2	Presence of two risk factors
Grade 3	Presence of three risk factors
* UCBT denotes unrelated cord blood transplantation.	

Supplementary Table 2. Demographic and clinical characteristics of patients in the retrospective study.

	UCBT patients (N=439)	PBSCT patients (N=162)
Median age (range), yr.	12 (1-70)	32 (1-62)
Median weight (range), kg	40 (8-100)	60 (14-97)
Male sex, n (%)	279 (63.6)	103 (63.6)
Sex (donor/recipient), n (%)		
Male/male	137 (31.2)	55 (34.0)
Male/female	85 (19.4)	37 (22.8)
Female/male	140 (31.9)	47 (29.0)
Female/female	73 (16.6)	22 (13.6)
Missing data	4 (0.9)	1 (0.6)
Diagnosis, n (%)		
Acute myeloid leukemia	156 (35.5)	59 (36.4)
Acute lymphoblastic leukemia	219 (49.9)	54 (33.3)
Myelodysplastic syndrome	27 (6.2)	18 (11.1)
Mixed lineage leukemia	4 (0.9)	2 (1.2)
Chronic myeloid leukemia	27 (6.2)	26 (16.0)
Lymphoma	6 (1.4)	2 (1.2)
Multiple myeloma	0 (0)	1 (0.6)
Conditioning regimen, n (%)		

TBI+CY+/-others	150 (34.2)	47 (29.0)
BU+CY+/-others	289 (65.8)	115 (71.0)
GVHD prophylaxis, n (%)		
CsA+MMF	433 (98.6)	114 (70.4)
CsA+MMF+MTX	6 (1.4)	34 (21.0)
CsA+MMF+ATG	0 (0)	4 (2.5)
CsA+MTX	0 (0)	3 (1.9)
CsA+MMF+CY	0 (0)	3 (1.9)
CsA+ATG	0 (0)	1 (0.6)
CsA+MMF+ATG+MTX	0 (0)	3 (1.9)

Abbreviations: TBI, total body irradiation; CY, cyclophosphamide; BU, busulfan; GVHD, graft-versus-host disease; CsA, cyclosporine A; MMF, mycophenolate mofetil; MTX, methotrexate; ATG, anti-thymocyte globulin.

Supplementary Table 3. Demographic and clinical characteristics of UCBT patients with and without PES.

	PES (N=335)	Non-PES (N=104)	p
Median age (range), yr.	12 (1-64)	14 (1-70)	
Median weight (range), kg	40 (9-82)	45 (8-100)	
Male sex, n (%)	200 (59.7)	79 (76.0)	
Diagnosis, n (%)			0.185
Acute myeloid leukemia	116 (34.6)	40 (38.5)	
Acute lymphoblastic leukemia	175 (52.2)	44 (42.3)	
Myelodysplastic syndrome	18 (5.4)	9 (8.7)	
Mixed lineage leukemia	4 (1.2)	0 (0)	
Chronic myeloid leukemia	19 (5.7)	8 (7.7)	
Lymphoma	3 (0.9)	3 (2.9)	
Conditioning regimen, n (%)			0.912
TBI+CY+/-others	114 (34)	36 (34.6)	
BU+CY+/-others	221 (66)	68 (65.4)	
GVHD prophylaxis, n (%)			0.148
CsA+MMF	332 (99.1)	101 (97.1)	
CsA+MMF+MTX	3 (0.9)	3 (2.9)	

Abbreviations: GVHD, graft-versus-host disease; TBI, total body irradiation; CY, cyclophosphamide; BU, busulfan; CsA, cyclosporine A; MMF, mycophenolate mofetil;

MTX, methotrexate.

Supplementary Table 4. Demographic and clinical characteristics of patients with severe PES in the single-arm study and the historical controls' study.

	The single-arm study (N=11)	The historical controls' study (N=50)	p
Median age (range), yr.	5 (2-22)	10 (1.5-50)	
Median weight (range), kg	19 (10-43)	32 (9-82)	
Male sex, n (%)	6 (54.5)	26 (52.0)	
Sex (donor/recipient),			0.188
Male/male	4 (36.4)	14 (28)	
Male/female	1 (9.1)	17 (34)	
Female/male	2 (18.2)	12 (24)	
Female/female	4 (36.4)	7 (14)	
Diagnosis, n (%)			0.27
Acute myeloid leukemia	4 (36.4)	14 (28)	
Acute lymphoblastic leukemia	4 (36.4)	30 (60)	
Myelodysplastic syndrome	2 (18.2)	4 (8)	
Chronic myeloid leukemia	1 (9.1)	1 (2)	
Lymphoma	0 (0)	1 (2)	
Conditioning regimen, n (%)			1
TBI+CY+/-others	2 (18.2)	8 (16)	
BU+CY+/-others	9 (81.8)	42 (84)	

GVHD prophylaxis, n (%)			0.18
CsA+MMF	10 (90.9)	50 (100)	
CsA+MMF+MTX	1 (9.1)	0 (0)	

Abbreviations: TBI, total body irradiation; CY, cyclophosphamide; BU, busulfan; GVHD, graft-versus-host disease; CsA, cyclosporine A; MMF, mycophenolate mofetil; MTX, methotrexate.

Supplementary Table 5. Antibodies used in this study.

Antibodies	SOURCE	IDENTIFIER	Dilution
Anti-human CD45 PerCP-CY5.5	Biolegend	Cat#368504	2 μl per test
Anti-human CD14 APC-CY7	BD Bioscience	Cat#557831	2 μl per test
Anti-human CD3 BV605	Biolegend	Cat#344836	0.5 μl per test
Anti-human CD56 BV421	Biolegend	Cat#318328	0.5 μl per test
Anti-human CD19 PE-CY7	Biolegend	Cat#302216	2 μl per test
Anti-human IFN-γ FITC	BD Bioscience	Cat#554700	2 μl per test
Anti-human IL-1β FITC	BD Bioscience	Cat#340515	2 μl per test
Anti-human IL-17A PE	eBioscience	Cat#12-7179-42	2 μl per test
Anti-human GM-CSF PE/Dazzle™ 594	Biolegend	Cat#502318	2 μl per test
Anti-human IL-6 FITC	Biolegend	Cat#501104	2 μl per test
Anti-human IL-6 PE	eBioscience	Cat#12-7069-82	2 μl per test
Anti-human TNF-α PE	Biolegend	Cat#502909	2 μl per test
Anti-human GM-CSFRα (CD116) PE	Biolegend	Cat#305908	2 μl per test
Anti-Rat IgG1, κ FITC	BD Bioscience	Cat# 554684	2 μl per test
Anti-mouse IgG1, κ FITC	BD Bioscience	Cat# 555748	2 μl per test
Anti- Rat IgG1, κ PE	BD Bioscience	Cat# 551979	2 μl per test
Anti-mouse IgG1, κ PE	BD Bioscience	Cat# 555749	2 μl per test
Anti-mouse IgG1, κ PerCP-CY5.5	BD Bioscience	Cat# 552834	2 μl per test
Anti-mouse IgG1, κ PE-CY7	BD Bioscience	Cat#557872	2 μl per test
Anti-mouse IgG2b, κ APC-CY7	BD Bioscience	Cat#558061	2 μl per test
Anti-mouse IgG1, κ BV421	Biolegend	Cat#406616	2 μl per test
Anti-mouse IgG1, κ BV605	Biolegend	Cat#400162	0.5 μl per test
Anti- Rat IgG2a, κ PE/Dazzle™ 594	Biolegend	Cat#400557	2 μl per test

Supplementary Table 6. Primers used in this study.

Oligonucleotides
Primers for human *GM-CSF*
Forward: AACTTCCTGTGCAACCCAGAT
Reverse: TTCTTCTGCCATGCCTGTATCA
Primers for human *IL-6*
Forward: TAC ATCCTCGACGGCATCTC
Reverse: AGCTCTGGCTTGTTCCTCAC
Primers for human *TNF*
Forward: GCCCATGTTGTAGCAAACCC
Reverse: TGATGGCAGAGAGGAGGTTG
Primers for human ACTIN
Forward: TTGCCGACAGGATGCAGAA
Reverse: GCCGATCCACACGGAGTACTT

Supplementary Table 7. Key reagents used in this study.

REAGENT	SOURCE	IDENTIFIER
Chemicals, Peptides, and Recombinant Proteins		
Human recombinant GM-CSF	Peprotech	Cat#AF-300-03-20
Human recombinant G-CSF	Peprotech	Cat#AF-300-23-10
LPS	Sigma	Cat#L2630-PMG
TRIzol reagent	Invitrogen	Cat#15596018
Critical Commercial Assays		
Foxp3/Transcription Factor Staining Buffer Set	Invitrogen	Cat#00-5523-00
FACS™ lysing solution	BD Biosciences	Cat#349202
M-MLV Reverse Transcriptase	Invitrogen	Cat#28025013
TB Green Premix Ex Taq II	TaKaRa	Cat#RR820
Anti-human CD14 MicroBeads	Miltenyi Biotec	Cat#130-050-201
Human IL-6 ELISA kits	R&D	Cat# D6050
Human IL-6 ELISA kits	Dakewe Biotech	Cat# 1110602
Human GM-CSF ELISA kits	R&D	Cat# HSGM0
Human TNF-α ELISA kits	Dakewe Biotech	Cat#1117202
Human IFN-γ ELISA kits	Dakewe Biotech	Cat#1110002
Human IL-1β ELISA kits	Dakewe Biotech	Cat#1110122
Human MCP-1 ELISA kits	Dakewe Biotech	Cat#1117392
Human IL-8 ELISA kits	Dakewe Biotech	Cat#1110802

Consort diagram

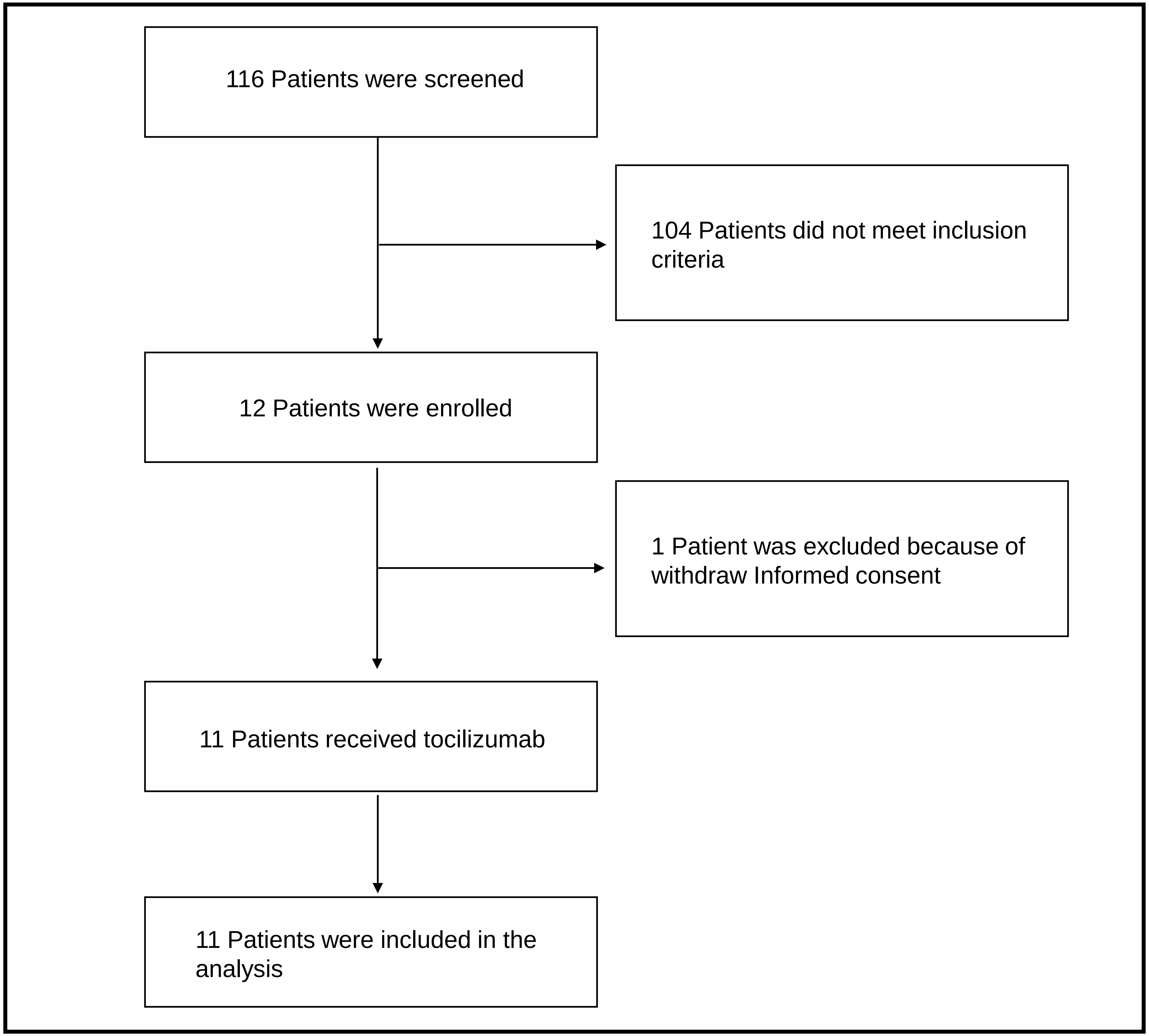

Trial profile. Flowchart of patients enrolled in the intervention trial.

Supplementary Note 1

A single arm, single-center clinical study of tocilizumab in the treatment of corticosteroids unresponsive pre-engraftment syndrome patients after unrelated cord blood transplantation

Registration number：ChiCTR1800015472

This is an English translation of the main sections of the original study protocol, the original protocol was approved by the Chinese Ethics Committee of Registering Clinical Trials, which is accessible at [http://www.chictr.org.cn], or from the corresponding author (ustcwhm@ustc.edu.cn) upon request.

1. Official title

A single arm, single-center clinical study of tocilizumab in the treatment of corticosteroids unresponsive pre-engraftment syndrome patients after unrelated cord blood transplantation

2. Participating Centers

Study participants were recruited from the First Affiliated Hospital of the University of Science and Technology in China (Anhui Provincial Hospital), Hefei, China.

3. Name and address of PI

Name of PI: Zimin Sun, M.D.

Address: Department of Hematology, The First Affiliated Hospital of USTC, Division of Life Sciences and Medicine, University of Science and Technology of China, 17 Lujiang Road, Hefei, China, Tel: 86-0551-62283347, Fax: 86-0551-62283347, E-mail: zmsun@ustc.edu.cn

4. Study design

Intervention model: Single group assignment

Masking: None (Open label)

5. Study Interventions

Biological: Tocilizumab

6. Study arms

Experimental: Single arm

Name: Tocilizumab immunotherapy

Dosage: 4 - 8 mg/kg

7. Brief summary

Pre-engraftment syndrome (PES) is common following UCBT and is characterized by non-infectious high-grade fever, skin rash, diarrhea and other clinical findings. At present, steroids treatment is the first-line therapy for PES. However, some patients are steroid-refractory. In our study, we found that IL-6 is a signature cytokine of PES, and to test the efficacy of this finding into a clinical setting, we conducted a single-arm trial to treat the patients who suffered steroid-refractory severe PES after a single-unit UCBT as a first HSCT. Eligible patients were treated with the anti-IL-6 receptor monoclonal antibody-tocilizumab. The study will evaluate if tocilizumab help to ameliorate PES, and also study the safety of treatment with tocilizumab.

8. Estimated enrollment

10 participants, if necessary, the sample size can be further expanded.

9. Study execution time

Study start date: April 2018

Estimated study completion date: April 2019

10. Study population

10.1 Inclusion criteria

(1) Patient was diagnosed with malignant hemaotogical diseases and needed UCBT according to Expert consensus on the treatment of hematological diseases by allogeneic hematopoietic stem cell transplantation in China;

(2) Myeloablative conditioning regimens, GVHD prophylaxis without ATG;

(3) Any age, any sex, any race;

(4) Must be met the diagnostic criteria of PES, if symptoms were not relieved after 3 days of methylprednisolone (2mg/kg/d) treatment, and the patient had a fever in excess of 38.3°C for three consecutive days;

(5) Patients and their families voluntarily participate in this research and sign informed consent.

10.2 Exclusion criteria

(1) Active infections, including bacteria, virus, fungi infection;

(2) No hypersensitivity to Tocilizumab or other accessories;

(3) Refuse to sign the infromed consent;

(4) Participate in other clinical experiments simultaneously.

11. Discontinuation of subjects from treatment

The treatment must be stopped for any one of the following reasons:

(1) Severe organ dysfunction during the research course;

(2) Withdrawal of informed consent for any reason;

12. Outcome measures

12.1 Primary Objective

Non-relapse mortality

12.2 Secondary Objectives

(1) Safety profile

(2) Neutrophil engraftment

(3) Platelet engraftment

(4) Overall survival

(5) Leukemia-free survival

13. Recruitment of subjects

(1) All patients meeting the inclusion criteria were recruited.

(2) All patients included in our study signed informed consent.

(3） Ineligible patients were given other treatment under the guidance of a doctor.

14. Baseline data of patients included:

Demography: name, sex, age, weight, diagnosis, body temperature, conditioning regimen, GVHD prophylaxis, number of HLA mismatch, ABO compatibility.

Laboratory tests: blood routine test, stool analysis, infused total nuclear cells (TNCs) and $CD34^+$ cells of cord blood, liver function, renal function, electrolyte, morphology, immunophenotyping.

15. Treatment protocols

All patients were treated following the standard UCBT procedures in our center [Zhu X, Huang L, Zheng C, et al: European group for blood and marrow transplantation risk score predicts the outcome of patients with acute leukemia receiving single umbilical cord blood transplantation. Biol Blood Marrow Transplant 23:2118-2126, 2017].

15.1 Cord blood selection

Cord blood and recipient HLA typing were determined using molecular techniques, with a minimum antigen split-level resolution for HLA-A and -B and allele-level resolution at DRB1. Cord blood units are from Chinese cord blood banks serologically matched for ≥4 of 6 HLA antigens and containing at least 2.5×10^7 TNC/kg and 1.2×10^5 $CD34^+$ cells/kg of recipient body weight before freezing were chosen for transplantation. Complete information regarding allele-level HLA matching for HLA-A, -B, -Cw, -DRB1 and -DQB1 loci was obtained from the Chinese cord blood banks.

15.2 Conditioning regimens

The myeloablative conditioning regimens were based on a full dose of busulfan (BU, total 12.8 mg/kg, 0.8 mg/kg every 6 hours for 4 days), cyclophosphamide (60 mg/kg daily for 2 days) plus fludarabine (30 mg/m^2 daily for 4 days) or total body irradiation (total 12 Gy, 3 cGy twice a day for two days) and cyclophosphamide (a total of 120 mg/kg administered as 60 mg/kg daily for 2 days) plus cytarabine (2 g/m2 twice a day for 2 days).

15.3 GVHD prophylaxis

All patients received a combination of cyclosporine (CsA) and mycophenolate mofetil (MMF) without antithymocyte globulin (ATG) as GVHD prophylaxis.

15.4 Cytokine supportive care

Granulocyte colony-stimulating factor (G-CSF; 5 to 7 μg/kg per day) was added on Day 6 after UCBT to stimulate neutrophil recovery.

15.5 Tocilizumab treatment

All recruited patients were given a comprehensive workup, including urine and blood cultures through both peripheral and central lines, and none of the patients responded to empirical antibiotic therapy. Consequently, these patients were treated with methylprednisolone (2mg/kg/d), if symptoms were not relieved after 3 days of methylprednisolone treatment, and the patient had a fever in excess of 38.3°C for three consecutive days, then tocilizumab was administered.

Eligible patients were treated with 4 - 8 mg/kg of tocilizumab (Tocilizumab Actemra, Roche). The clinical trial was registered at www.chictr.org.cn (Reference Number: ChiCTR1800015472). This study was approved by the Chinese Ethics Committee of Registering Clinical Trials (Reference Number: ChiECRCT-20180129). All subjects provided written informed consent in order to participate in the study.

16. Definitions and evaluation

(1) The date of cord blood infusion was defined as day 0.

(2) The date of neutrophil engraftment was defined as the first day of a neutrophil count $\geq 0.5 \times 10^9$/L for three consecutive days.

(3) The date of platelet engraftment was defined as the first day of platelet recovery to $\geq 20 \times 10^9$/L without transfusion support for seven consecutive days.

(4) Acute GVHD was defined and scored from 0 to IV, according to the type and severity of organ involvement. [Przepiorka D, Weisdorf D, Martin P, et al: 1994 Consensus Conference on Acute GVHD Grading. Bone Marrow Transplant 15:825-828, 1995]; grades III-IV aGVHD is characterized by severe clinical features of the skin,

liver and/or gut.

(5) Chronic GVHD was defined and graded according to the National Institutes of Health criteria [Jagasia MH, Greinix HT, Arora M, et al: National Institutes of Health consensus development project on criteria for clinical trials in chronic graft-versus-host disease: I. The 2014 Diagnosis and Staging Working Group report. Biol Blood Marrow Transplant 21: 389-401, 2015]. According to these criteria, mild cGVHD reflects one or two organs involved with no more than score 1 plus lung score 0; moderate cGVHD involves three or more organs involved with no more than score 1 or at least one organ (not lung) with a score of 2 or lung score 1; and extensive cGVHD is diagnosed when at least one organ with a score of 3 or lung score of 2 or 3. The diagnosis was mainly based on clinical manifestations.

(6) Relapse was defined by the morphological evidence of disease in the peripheral blood, BM or extramedullary sites. Time to relapse was defined from the date of transplantation to the date of disease recurrence. Patients exhibiting minimal residual disease (for example, the presence of BCR/ABL RNA transcripts by PCR) were not classified as having relapsed.

(7) Overall survival was calculated from day 0 to the time of death or last follow-up.

(8) Disease-free survival (DFS) was defined as the time from day 0 to the date of disease progression or death from any cause.

(9) Transplant-related mortality was defined as all causes of death other than those related directly to malignant disease itself, occurring at any time after transplantation.

17. Safety profile

Safety analyses were performed based on the incidences of adverse events, transplant-related complications.

17.1 Adverse events

(1) Definition:

Adverse events refer to an unforeseeable medical condition during or after treatment or an event worsening health conditions that does not necessarily have a causal relationship with the treatment regimen used. Adverse events include symptoms, signs and abnormal laboratory results.

(2) Recording and grading:

Adverse events were recorded and graded according to the Common Terminology Criteria for Adverse Events, Version 4.0.

(3) Monitoring:

All patients were monitored by daily physical examination and routine blood tests prior to neutrophil. Liver and kidney function tests were performed at least twice a week within 30 days after UCBT. After neutrophil engraftment, serum cytomegalovirus (CMV)-DNA was also detected once a week until day 90.

17.2 Transplant-related complications

(1) Bleeding events were the major transplant-related complication after UCBT.

(2) Grading:

The assessment of bleeding events, with the exception of mild petechiae, was performed during the first 60 days post-UCBT according to previously published criteria [Labrador J, Lopez-Anglada L, Perez-Lopez E, et al: Analysis of incidence, risk factors and clinical outcome of thromboembolic and bleeding events in 431 allogeneic hematopoietic stem cell transplantation recipients. Haematologica 98:437-443, 2013]. Bleeding severity was graded as minor, major-nonlife-threatening and major-life-

threatening. Major bleeding was defined if it induced a reduction in the hemoglobin level of at least 20 g/L, transfusion of at least two blood-pack units, or symptomatic bleeding in a critical area or organ. Transfusion events due to aplasia post chemotherapy were not included. Major bleeding was considered to be life-threatening if it resulted in fatality, symptomatic intracranial or pulmonary bleeding, bleeding with a decrease in the hemoglobin level of at least 50 g/L, bleeding requiring the transfusion of at least four red blood-cell units or inotropic agents, or surgery. All other bleeding was considered minor.

18. Statistical analysis

Numerical variables were described as medians and compared using the Mann–Whitney U test, and Pearson's chi-square test was used for categorical variables. Cumulative incidence curves were used in a competing-risks setting, to calculate the probability of neutrophil engraftment, platelet engraftment and recovery, acute GVHD, TRM, and relapse. The occurrence of death prior to neutrophil engraftment, platelet engraftment and platelet recovery was considered a competing risk. Competing risks for the occurrence of GVHD were death without GVHD and relapse without GVHD. The competing risk for TRM was death with relapse. When relapse of leukemia was the outcome of interest among patients with this disease, death without relapse was a competing risk event. The probabilities of OS and DFS were estimated using the Kaplan-Meier method.

All statistical analyses were performed using R software package (version 3.5.0), Prism 6 (GraphPad) and SPSS 25.0 software, and P-values less than 0.05 were considered statistically significant.

Supplementary Note 2

Multicenter clinical study of the risk classification and stratified intervention on pre-engraftment syndrome after umbilical cord blood transplantation

Registration number：ChiCTR-ONC-16009013

1. Title of study

Multi center clinical study of the risk classification and stratified intervention on pre-engraftment syndrome after umbilical cord blood transplantation

2. Objectives of Study

Further verification for hematologic malignancies patients with PES after umbilical cord blood transplantation, according to the PES dangerous degree classification using different doses of MP stratified intervention treatment can timely and effective control of PES, and further improve the prognosis of patients with severe PES without affecting cord blood engraftment.

3. Hospitals included

The First Affiliated Hospital of the University of Science and Technology in China,
The Third Affiliated Hospital of Sun-yat sen University,
Wangnan Medical College yijishan hospital.

4. Name and address of PI

Name of PI: Zimin Sun, M.D.

Address: Department of Hematology, The First Affiliated Hospital of USTC, Anhui Provincial Hospital, Division of Life Sciences and Medicine, University of Science and Technology of China, 17 Lujiang Road, Hefei, China, Tel: 86-0551-62283347, Fax: 86-0551-62283347, E-mail: zmsun@ustc.edu.cn

5. Study population

5.1 Inclusion criteria

(1) Diagnosed with hematologic malignancies, any age, any gender, any race;

(2) No HLA matched sibling donor or unwilling to choose HLA matched sibling donor transplantation;

(3) No severe organ failure and active infection;

(4) Patients and their families voluntarily carried out UCBT and signed informed consent;

(5) If PES occurs, patients and their families voluntarily carry out different doses of MP

stratified intervention treatment and signed informed consent.

5.2 Exclusion criteria

(1) Serious viscera dysfunction or disease, such as serious diseases and function disorder in heart, liver, kidney and pancreas;

(2) Patients with pregnancy;

(3) Participants and/or authorized family members refused to accept UCBT;

(4) Researchers believe that can damage the unnecessary risks to safety, make the results of the study subjects any life-threatening illness, condition or organ system dysfunction; drug dependence; uncontrolled mental disease; cognitive impairment;

(5) Participate in other similar clinical researches in 3 months;

(6) Researchers think that the patient doesn't fit into the group (e.g., patients are expected to be discontinue because of funding problems).

6. outcomes

6.1 primary outcomes

180d transplant related mortality

6.2 Secondary outcomes

(1) Cumulative incidence of acute GVHD

(2) Relapse rate

(3) One-year overall survival

(4) One-year leukemia free survival

(5) Cumulative rate of neutrophil engraftment

(6) Neutrophil engraftment time

(7) Cumulative rate of platelet engraftment

(8) Platelet engraftment time
