# Supplementary material for: Inflammatory monocytes promote pre-engraftment syndrome and tocilizumab can therapeutically limit pathology in patients
Source: Nat Commun. 2021 Jul 6;12:4137. doi: 10.1038/s41467-021-24412-1 (PMC8260612; doi:10.1038/s41467-021-24412-1)
Supplement: Supplementary file 3 — Reporting Summary [file 41467_2021_24412_MOESM3_ESM.pdf]

nature research

Corresponding author(s): Haiming Wei, Zhigang Tian, Zimin Sun

Last updated by author(s): May 18, 2021

Reporting Summary

Nature Research wishes to improve the reproducibility of the work that we publish. This form provides structure for consistency and transparency in reporting. For further information on Nature Research policies, see our Editorial Policies and the Editorial Policy Checklist.

Statistics

For all statistical analyses, confirm that the following items are present in the figure legend, table legend, main text, or Methods section.

n/a	Confirmed	
☐	☒	The exact sample size (*n*) for each experimental group/condition, given as a discrete number and unit of measurement
☐	☒	A statement on whether measurements were taken from distinct samples or whether the same sample was measured repeatedly
☐	☒	The statistical test(s) used AND whether they are one- or two-sided<br>*Only common tests should be described solely by name; describe more complex techniques in the Methods section.*
☐	☒	A description of all covariates tested
☐	☒	A description of any assumptions or corrections, such as tests of normality and adjustment for multiple comparisons
☐	☒	A full description of the statistical parameters including central tendency (e.g. means) or other basic estimates (e.g. regression coefficient) AND variation (e.g. standard deviation) or associated estimates of uncertainty (e.g. confidence intervals)
☐	☒	For null hypothesis testing, the test statistic (e.g. *F*, *t*, *r*) with confidence intervals, effect sizes, degrees of freedom and *P* value noted<br>*Give P values as exact values whenever suitable.*
☒	☐	For Bayesian analysis, information on the choice of priors and Markov chain Monte Carlo settings
☒	☐	For hierarchical and complex designs, identification of the appropriate level for tests and full reporting of outcomes
☒	☐	Estimates of effect sizes (e.g. Cohen's *d*, Pearson's *r*), indicating how they were calculated

Our web collection on statistics for biologists contains articles on many of the points above.

Software and code

Policy information about availability of computer code

Data collection	The Flow cytometric data: LSR II and LSRFortessa flow cytometer (BD Biosciences)<br>The R software package (version 3.5.0)<br>The SPSS 25.0 software<br>The Prism 6 (GraphPad)
Data analysis	Statistical analysis: R software package (version 3.5.0), GraphPad Prism (ver. 6) and SPSS 25.0 software.<br>Flow cytometric analysis: FlowJo (Version 10.0.7 for Windows)<br>Microarray analyses: Feature Extraction v10.7.1.1 (Agilent Technologies) and GeneSpring GX (Agilent Technologies)

For manuscripts utilizing custom algorithms or software that are central to the research but not yet described in published literature, software must be made available to editors and reviewers. We strongly encourage code deposition in a community repository (e.g. GitHub). See the Nature Research guidelines for submitting code & software for further information.

Data

Policy information about availability of data

All manuscripts must include a data availability statement. This statement should provide the following information, where applicable:

- Accession codes, unique identifiers, or web links for publicly available datasets
- A list of figures that have associated raw data
- A description of any restrictions on data availability

The main data that support the findings of this study are available in the article and its Supplementary Information. Microarray data were deposited into the National Center for Biotechnology Information GEO repository (accession number: GSE128562). The data that support the findings of this study are available from the corresponding author upon request.

Field-specific reporting

Please select the one below that is the best fit for your research. If you are not sure, read the appropriate sections before making your selection.

- [x] Life sciences
- [ ] Behavioural & social sciences
- [ ] Ecological, evolutionary & environmental sciences

For a reference copy of the document with all sections, see nature.com/documents/nr-reporting-summary-flat.pdf

Life sciences study design

All studies must disclose on these points even when the disclosure is negative.

Sample size	For the experimental part, this sample size was sufficient to demonstrate statistically significant differences in comparisons between experimental groups by two-tailed Student t-test and ANOVA test and Mann-Whitney test. The sample size was also determined to be adequate based on the reproducibility between independent experiments. The clinical trial was a pilot study, sample size calculations are not performed in this study. The data obtained in this study will provide information for future sample size calculations in large studies.
Data exclusions	No data were excluded.
Replication	All experiments were either successfully replicated using a sufficient human sample size. Representative data was confirmed at least three times.
Randomization	This single arm clinical study was applied and randomization was not relevant.
Blinding	This single arm clinical study was applied and blinding was neither relevant nor possible

Reporting for specific materials, systems and methods

We require information from authors about some types of materials, experimental systems and methods used in many studies. Here, indicate whether each material, system or method listed is relevant to your study. If you are not sure if a list item applies to your research, read the appropriate section before selecting a response.

Materials & experimental systems

n/a	Involved in the study	
[ ]	[x]	Antibodies
[x]	[ ]	Eukaryotic cell lines
[x]	[ ]	Palaeontology and archaeology
[x]	[ ]	Animals and other organisms
[ ]	[x]	Human research participants
[ ]	[x]	Clinical data
[x]	[ ]	Dual use research of concern

Methods

n/a	Involved in the study	
[x]	[ ]	ChIP-seq
[ ]	[x]	Flow cytometry
[x]	[ ]	MRI-based neuroimaging

Antibodies

Antibodies used

nti-human CD45 PerCP-CY5.5, Biolegend, Cat#368504; 2μl per test;
Anti-human CD14 APC-CY7, BD Bioscience, Cat#557831; 2μl per test;
Anti-human CD3 BV605, Biolegend, Cat#344836; 0.5μl per test;
Anti-human CD56 BV421, Biolegend, Cat#318328; 0.5μl per test;
Anti-human CD19 PE-CY7, Biolegend, Cat#302216; 2μl per test;
Anti-human IFN-γ FITC, BD Bioscience, Cat#554700; 2μl per test;
Anti-human IL-1β FITC, BD Bioscience, Cat#340515; 2μl per test;

Anti-human IL-17A PE, eBioscience, Cat#12-7179-42; 2µl per test;
Anti-human GM-CSF PE/Dazzle™ 594, Biolegend, Cat#502318; 2µl per test;
Anti-human IL-6 FITC, Biolegend, Cat#501104; 2µl per test;
Anti-human IL-6 PE, eBioscience, Cat#12-7069-82; 2µl per test;
Anti-human TNF-α PE, Biolegend, Cat#502909; 2µl per test;
Anti-human GM-CSFRα (CD116) PE, Biolegend, Cat#305908; 2µl per test;
Anti-Rat IgG1, κ FITC, BD Bioscience, Cat# 554684; 2µl per test;
Anti-mouse IgG1, κ FITC, BD Bioscience, Cat# 555748; 2µl per test;
Anti- Rat IgG1, κ PE, BD Bioscience, Cat# 551979; 2µl per test;
Anti-mouse IgG1, κ PE, BD Bioscience, Cat# 555749; 2µl per test;
Anti-mouse IgG1, κ PerCP-CY5.5, BD Bioscience, Cat# 552834; 2µl per test;
Anti-mouse IgG1, κ PE-CY7, BD Bioscience, Cat#557872; 2µl per test;
Anti-mouse IgG2b, κ APC-CY7, BD Bioscience, Cat#558061; 2µl per test;
Anti-mouse IgG1, κ BV421, Biolegend, Cat#406616; 0.5µl per test;
Anti-mouse IgG1, κ BV605, Biolegend, Cat#400162; 0.5µl per test;
Anti- Rat IgG2a, κ PE/Dazzle™ 594, Biolegend, Cat#400557 2µl per test;.

Validation

All antibodies are commercially available and were commercially validated

Human research participants

Policy information about studies involving human research participants

Population characteristics

11 severe PES patients received tocilizumab and were evaluable. The age of these 11 patients range from 2 to 22 and the median age was 5, the weight of these patients range from 10 to 43 and the median weight was 19, including 6 males and 5 females. All patients underwent modified myeloablative conditioning chemotherapy or chemoradiotherapy. Nine patients received fludarabine (30 mg/m2 for 4 days), busulfan (a total of 12.8 mg/kg, along with 0.8 mg/kg of intravenous busulfan every 6 hours for 4 days) plus 60 mg/kg of cyclophosphamide for 2 days. Two patients received total body irradiation (a total of 12 cGy; 3 cGy twice a day for two days), cyclophosphamide (a total of 120 mg/kg administered as 60 mg/kg daily for 2 days) and cytarabine (2 g/m2 twice a day for 2 days).

Recruitment

Inclusion criteria:
1. Patient was diagnosed with malignant hemaotogical diseases and needed UCBT according to Expert consensus on the treatment of hematological diseases by allogeneic hematopoietic stem cell transplantation in China;
2. Myeloablative conditioning regimens, GVHD prophylaxis without ATG;
3. Any age, any sex, any race;
4. Must be met the diagnostic criteria of PES, if symptoms were not relieved after 3 days of methylprednisolone (2 mg/kg) treatment, and the patient had a fever in excess of 38.3خ for three consecutive days;
5. Patients and their families voluntarily participate in this research and sign informed consent.
Exclusion criteria:
1. Active infections, including bacteria, virus, fungi infection;
2. No hypersensitivity to Tocilizumab or other accessories;
3. Refuse to sign the infromed consent;
4. Participate in other clinical experiments simultaneously.

Ethics oversight

This study was approved by the Chinese Ethics Committee of Registering Clinical Trials (Reference Number: ChiECRCT-20180129). All subjects or their guardians provided written informed consent in order to participate in the study.

Note that full information on the approval of the study protocol must also be provided in the manuscript.

Clinical data

Policy information about clinical studies

All manuscripts should comply with the ICMJEguidelines for publication of clinical research and a completedCONSORT checklist must be included with all submissions.

Clinical trial registration

The clinical trial was registered at www.chictr.org.cn (Reference Number: ChiCTR1800015472).

Study protocol

The clinical trial protocol can be accessed. An English translation of the main sections (including inclusion/exclusion criteria, and pre-specified outcomes) of the original study protocol is available within the Supplementary Information file.

Data collection

The following patient characteristics were obtained:
- age, sex, weight, diagnosis, conditioning regimen, body temperature;
- blood routine test, the dosage of tocilizumab.

Outcomes

The primary outcome is non-relapse mortality. Relapse was defined by the morphological evidence of disease in the peripheral blood, BM or extramedullary sites. Time to relapse was defined from the date of transplantation to the date of disease recurrence. Patients exhibiting minimal residual disease (for example, the presence of BCR/ABL RNA transcripts by PCR) were not classified as having relapsed.

The secondary outcomes included neutrophil engraftment, platelet engraftment and overall survival. The date of neutrophil engraftment was defined as the first day of a neutrophil count ≥0.5 ×109/L for three consecutive days.The date of platelet engraftment was defined as the first day of platelet recovery to ≥20×109/L without transfusion support for seven consecutive days. Overall survival was calculated from the day of transplantation to the time of death or last follow-up. OS was assessed using Kaplan-Meier approach.

Flow Cytometry

Plots

Confirm that:

- [x] The axis labels state the marker and fluorochrome used (e.g. CD4-FITC).
- [x] The axis scales are clearly visible. Include numbers along axes only for bottom left plot of group (a 'group' is an analysis of identical markers).
- [x] All plots are contour plots with outliers or pseudocolor plots.
- [x] A numerical value for number of cells or percentage (with statistics) is provided.

Methodology

Sample preparation

Mononuclear cells were isolated from umbilical cord blood (The First Affiliated Hospital of University of Science and Technology of China) and prepared from buffy coats obtained from healthy infant donors by centrifugation and a Ficoll system. Cord blood was layered on top of Ficoll (tbd science, # LTS1077) in a 50 mL tube and centrifuged at 500 x g for 30 min at room temperature. Buffy coats were collected, washed twice in phosphate-buffered saline (PBS), and finally resuspended. Peripheral blood stem cells were collected from the peripheral blood through a process known as apheresis. Monocytes were purified by a magnetic-activated cell sorter (MACS) kit (Miltenyi Biotec, Germany). Detail of cell culture was described in Methods.

Instrument

The Flow cytometric data were collected by LSR II and LSRFortessa flow cytometer (BD Biosciences).

Software

Data were analysed with FlowJo (Version 10.0.7 for Windows)

Cell population abundance

Monocytes were purified by a MACS kit (CD14 MicroBeads, human, Miltenyi, # 130-050-201). The purity was higher than 90% as determined by secondary flow-cytometric analysis.

Gating strategy

For all experiments, cells were first gated by CD45/SSC to select leukocytes, followed by gating FSC-A and FSC-H to eliminate non-singlets. Then, target cell population for further analysis were gated by cell surface marker.

- [x] Tick this box to confirm that a figure exemplifying the gating strategy is provided in the Supplementary Information.
